# Sleep Deprivation’s Effect on Circadian Genes Expression and Its Associtations with Depression Symtoms and Psychomotor Abilities

**DOI:** 10.1192/j.eurpsy.2025.570

**Published:** 2025-08-26

**Authors:** A. Witkowska, A. Gabryelska, M. Ditmer, A. Tarasiuk-Zawadzka, A. Binienda, S. Turkiewicz, F. F. Karuga, P. Białasiewicz, J. Fichna, M. Sochal

**Affiliations:** 1 Department of Sleep Medicine and Metabolic Disorders; 2 Department of Biochemistry, Medical Univeristy of Lodz, Łódź, Poland

## Abstract

**Introduction:**

Sleep deprivation (SD) is a global health concern that impairs cognitive and psychomotor functions (PF). While it can temporarily improve mood, its effects on mood and connection to depressive symptoms (DS) remain unclear. These impacts may involve circadian rhythm gene regulation, though distinct evidence from human studies is lacking.

**Objectives:**

To assess the impact of SD on the expression of circadian rhythm regulation genes and its associations with the alleviation of DS. Additionally, to explore the relationship between changes in gene expression and PF following SD.

**Methods:**

Participants (n = 72) underwent a baseline sleep assesment by polysomnography (PSG), later being subjected to SD. In total, evaluation of mood And cognitive functions (Bimanual Eye-Hand Coordination Test - BEHCT) was conducted four times, pre/post PSG and SD. Moreover, circadian rhythm regulation genes expression: Circadian Locomotor Output Cycles Kaput (CLOCK), Brain and muscle Arnt-like protein-1 (BMAL1), Period Circadian Regulator 1 (PER1), Cryptochrome Circadian Regulator 1 (CRY1), Nuclear Receptor Subfamily 1 Group D Member 1 (NR1D1) and Neuronal PAS Domain Protein 2 (NPAS2) was evaluated. Participants were divided into respondents (RE, n = 49) and non-respondents (NR, n = 23) depending on changes in DS under the influence of SD by the Montgomery–Åsberg Depression Rating Scale evalluation.

**Results:**

No relationship was found between BEHCT parameters and the studied genes in the entire study group. NRs exhibited a negative correlation in number of motor function errors in relation to all examined genes of CLOCK (r=-0.52, p=0.02), BMAL1(r=-0.55, p=0.007), CRY1(r=-0.45, p=0.048), PER1(r=-0.6, p=0.023), and NR1D1 (r=-0.19, p=0.523) is except for NPAS2. Additionally, In NRs, the BEHCT error time negatively correlated with the PER1 and NR1D1 (r=-0.6, p=0.006; r=-0.52, p=0.045; respectively). In contrast, within the RE group, only NPAS2 expression showed a positive correlation with the number of errors (r= 0.35, p=0.049).

**Conclusions:**

Reduced expression of CLOCK, BMAL1, CRY1, PER1, and NR1D1 is associated with impaired PF, only in individuals with worsening DS after SD. Increased NPAS2 expression appears to be an orgin of reduced PF results in the RE group. These genes, integral to the circadian system’s feedback loop, may mediate the complex effects of SD on mood and cognitive function, warranting further investigation.

**Disclosure of Interest:**

None Declared

